# Impact of Three-Dimensional Strain on Major Adverse Cardiovascular Events after Acute Myocardial Infarction Managed by Primary Percutaneous Coronary Intervention—A Pilot Study

**DOI:** 10.3390/life11090930

**Published:** 2021-09-07

**Authors:** Raluca Tomoaia, Ruxandra Ștefana Beyer, Dumitru Zdrenghea, Alexandra Dădârlat-Pop, Mircea Ioachim Popescu, Gabriel Cismaru, Gabriel Gușetu, Adela Mihaela Șerban, Gelu Radu Simu, Ioan Alexandru Minciună, Bogdan Caloian, Radu Roșu, Maria Ioana Chețan, Dana Pop

**Affiliations:** 1 Cardiology Department, Heart Institute “N. Stăncioiu”, 400001 Cluj-Napoca, Romania; anda_bogdan@yahoo.com (R.Ș.B.); dadarlat.alexandra@yahoo.ro (A.D.-P.); adelamserban@yahoo.com (A.M.Ș.); ioana.chetan@umfcluj.ro (M.I.C.); 2 5th Department of Internal Medicine, Faculty of Medicine, “Iuliu Hațieganu” University of Medicine and Pharmacy, 400012 Cluj-Napoca, Romania; dzdrenghea@yahoo.com (D.Z.); cismaru.gabriel@umfcluj.ro (G.C.); gusetu@gmail.com (G.G.); simu.gelu@umfcluj.ro (G.R.S.); iaminciuna@gmail.com (I.A.M.); caloian.bogdan@umfcluj.ro (B.C.); ovidiu.rosu@umfcluj.ro (R.R.); pop67dana@gmail.com (D.P.); 3 Cardiology Department, Rehabilitation Hospital, 400347 Cluj-Napoca, Romania; 4 Department of Medical Disciplines, Faculty of Medicine and Pharmacy, University of Oradea, 410073 Oradea, Romania; procardia_oradea@yahoo.com; 5 Cardiology Department, Clinical County Emergency Hospital of Oradea, 410169 Oradea, Romania

**Keywords:** acute myocardial infarction, three-dimensional speckle-tracking, major adverse cardiovascular events, prognosis

## Abstract

Background: Three-dimensional speckle-tracking echocardiography (3D-STE) allows simultaneous assessment of multidirectional components of strain. However, there are few data on its usefulness to predict prognosis in patients with acute myocardial infarction (AMI). The objective of our pilot study was to evaluate the prognostic value of four different 3D-STE parameters (global longitudinal strain (GLS-3D), global circumferential strain (GCS-3D), global radial strain (GRS-3D), and global area strain (GAS)) in AMI, after successful revascularization by primary PCI. Methods: We enrolled 94 AMI patients (66 ± 13 years, 56% men) who underwent coronary angiography. All patients had been 3D-STE assessed and followed-up for 1 year for the occurrence of MACE. Results: A total of 25 MACE were recorded over follow-up. Cut-off values of −17% for GAS (HR = 3.1, 95% CI: 1.39–6.92, *p* = 0.005), −12% for GCS-3D (HR = 3.06, 95% CI: 1.36–6.8, *p* = 0.006), −10% for GLS-3D (HR = 3.04, 95% CI: 1.36–6.78, *p* = 0.006), and 25% for GRS-3D (HR = 2.89, 95% CI: 1.29–6.46, *p* = 0.009) showed moderate accuracy in MACE prediction. Multivariate regression showed that GAS (HR = 1.1, 95% CI: 1.03–1.16), GLS-3D (HR = 1.13, 95% CI: 1.03–1.26), and GCS-3D (HR = 1.13, 95% CI: 1.03–1.23) remained independent predictors of MACE (HR = 1.07, 95% CI: 1.01–1.14 for GAS, and HR = 1.1, 95% CI: 1.01–1.2 for GCS-3D). However, post hoc power analysis indicated adequate sample size (power of 80%) only for GAS and GCS-3D for the ROC curve analysis and for GAS, GCS-3D, and GRS-3D for the log-rank test. Conclusion: Patients with AMI might benefit from early risk stratification with the aid of 3D-STE measurements, particularly GAS and GCS-3D, but larger studies are necessary to determine the optimal cut-off values to predict MACE.

## 1. Introduction

The increased use of reperfusion strategies consisting of primary percutaneous coronary intervention (PCI) and antithrombotic therapy has improved survival in patients with acute myocardial infarction (AMI). However, major adverse cardiovascular events (MACE) are an important cause of morbidity and mortality in patients undergoing revascularization. Left ventricular ejection fraction (LVEF) has been the conventional parameter most used in the prediction of long-term post-AMI outcomes in these patients and has been incorporated into current guidelines [1,2]. However, LVEF remains controversial due to limitations caused by the operator experience, load dependency, and by ignoring the complex geometry of the left ventricle (LV) [3,4].

Two-dimensional speckle-tracking echocardiography (2D-STE) imaging overcomes geometric assumptions that might limit the use of standard LVEF and provides a detailed analysis of myocardial deformation, leading therefore to a better understanding of cardiac mechanics [5,6]. Both global longitudinal (GLS) and circumferential (GCS) strains have demonstrated a prognostic role after AMI and might be superior to standard LVEF in the prediction of cardiac events [7,8]. However, as myocardial mechanics involves multidirectional axes of motion, 2D-STE measurements are limited by the tracking of the speckles in a single plane. Further limitations include the necessity of multiple acquisitions in order to assess different directional components (three apical and three parasternal views) and the errors caused by heart variability and out-of-plane motion of the speckles [9,10].

Three-dimensional STE (3D-STE) is a recently developed echocardiographic technique allowing full 3D volume acquisitions of the LV, with simultaneous assessment of multidirectional components of strain and a single post-processing algorithm [9]. First, this technique saves time by allowing the calculation of all 3D-STE parameters from a single dataset. Secondly, it avoids errors caused by irregular heart rhythms. Thirdly, it offers a new parameter, global area strain (GAS), which combines the circumferential and longitudinal strain of the LV and dynamic segmental strain [10]. However, 3D-STE is limited by the dependency of image quality and by temporal and spatial resolution.

Recently, there have been a few studies reporting the predictive value of 3D-STE in patients with AMI [11,12,13,14]. The most significant results were obtained for GAS, which was independently associated with increased risk of MACE after AMI [11,12,14]. However, after AMI, myocardial layers may be variably affected depending on the degree of ischemia. It is thus necessary to determine multiple strain parameters corresponding to different types of myocardial mechanics in order to reflect the impact on MACE.

The objective of our pilot study was to evaluate the role of four different 3D-STE parameters (global longitudinal strain (GLS-3D), global circumferential strain (GCS-3D), global radial strain (GRS-3D), and global area strain (GAS)) in the prediction of clinical outcomes after successful revascularization of AMI by primary PCI.

## 2. Materials and Methods

### 2.1. Study Population

This study was conducted according to the guidelines of the Declaration of Helsinki and approved by Ethics Committee of “Iuliu Hațieganu” University of Medicine and Pharmacy, Cluj-Napoca under number 250 on 13 July 2020. We prospectively enrolled 110 patients with AMI, which were admitted in our Cardiology Department from July to August 2019. The diagnosis and treatment of AMI were performed in compliance with the European Guidelines of ST- and non-ST-elevation myocardial infarction (STEMI and NSTEMI) [1,2]. Patients with a previous MI, more than moderate valvular heart disease or moderate pulmonary hypertension, hypertrophic cardiomyopathy, and significant renal impairment (eGFR < 15 mL/min/1.73 m^2^) were excluded. All remaining patients underwent coronary angiography and were successfully revascularized by primary PCI. PCI was performed in patients with multivessel coronary artery disease (CAD) in later sessions. Echocardiography using traditional and 3D strain parameters was performed within the first 48 h after coronary angiography.

### 2.2. Echocardiography

**Figure 1:**
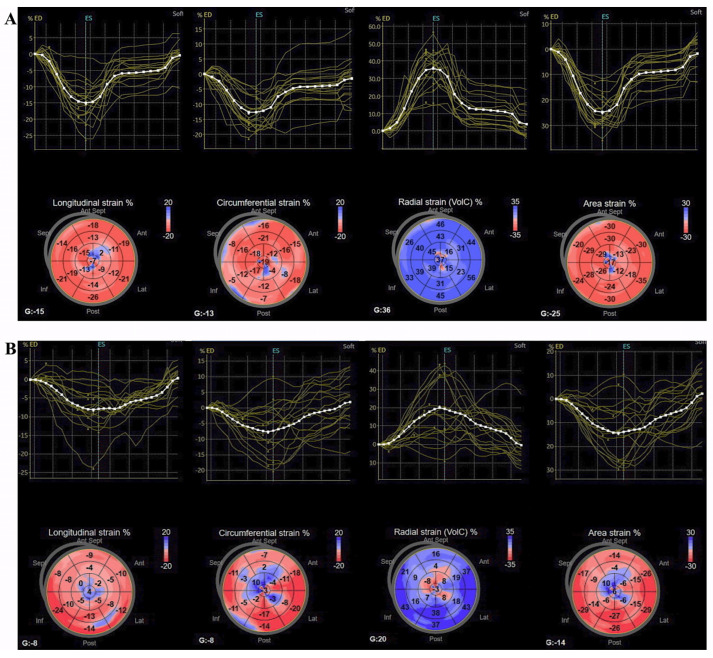
Representative cases of 3D-STE analysis. 3D strain parameters from two patients with anterior acute myocardial infarction and a LVEF of 48% (**A**) and of 42% (**B**), showing reduced strain values in the apical segments: GLS-3D, GCS-3D, GAS (negative, red colour), and GRS-3D (positive, blue colour) were simultaneously obtained. In the left bottom corner of each bull’s eye map, the global value is displayed for each parameter; 3D, three-dimensional; GAS, global area strain; GCS, global circumferential strain; GLS, global longitudinal strain; and GRS, global radial strain.

The echocardiography was performed on a Vivid E95 scanner (GE Vingmed Ultrasound, Norway) by using a real-time 3D phased-array transducer (4V-D) and analysed offline using EchoPac BT13 software (GE Vingmed Ultrasound). The LV end-diastolic volume (EDV) and end-systolic volume (ESV) were measured using a 2D matrix array probe (M5S) and LVEF was calculated using the modified Simpson’s biplane formula according to the current recommendations. Diastolic dysfunction parameters (trans-mitral peak early velocity, average e’ by tissue Doppler imaging, maximum tricuspid regurgitation velocity and left atrial volume index) were also measured. In this pilot study, the 4V-D probe was used to record a 3D full volume data set of the LV over four cardiac cycles at a frame rate of 25 to 30 frames/second. The acquired 3D datasets were transferred to a workstation for offline analysis. 3D-STE parameters were calculated using the 4D LV quantification function of the system (4D-autoLVQ). After the three apical long axis and short axis views alignment in order to minimize foreshortening, topographic markers were manually placed at the level of the mitral annulus and LV apex. The contour for the end-diastolic and end-systolic borders of the LV was automatically delineated and manual adjustment was performed where necessary; consequently, the automatic alignment of endocardial and epicardial contours was followed by manual adjustment, confirming the myocardial wall, where necessary, in order for all segments to be included in the strain analysis. The software automatically calculated mean peak systolic strain values before the end-systolic frame for GLS-3D, GCS-3D, GRS-3D, and GAS, and the LV mass. Tracked areas, bull’s eye diagrams, and tracking curves for each of the strain components were saved (Figure 1).

### 2.3. Interobserver Variability

As to evaluate the reproducibility of the 3D-STE measurements, we have randomly selected 20 patients for whom measurements were repeated by a second operator, who was blinded to the results of the first operator. We calculated the inter-observer correlation coefficients for GLS-3D, GCS-3D, GRS-3D, and GAS.

### 2.4. Follow-Up

The patients were evaluated at 1 year after first admission, by telephone or hospital record registration, in order to assess clinical symptoms. Clinical endpoints (MACE) were defined as heart failure (HF) requiring hospitalization, recurrent MI, repeat revascularization, and cardiac death. Hospitalization and death by non-cardiac cause were not considered events.

### 2.5. Statistics

Variables were expressed as the mean ± SD, median (IQR) or frequencies according to the type and distribution of the data. Normality was tested using the Kolmogorov–Smirnov test. We categorized the patients according to MACE presence or absence. Baseline parameters were compared using *t*-tests or Mann–Whitney U test according to data distribution of continuous data and chi^2^ test for categorical data. As there are no currently established cut-off values for 3D strain parameters, as to determine the role in risk stratification for 1-year MACE of each parameter, we used the cut-off values derived from Receiver Operating Characteristic (ROC) curves. We performed the ROC analysis with a bootstrap Youden index confidence interval and we obtained the 95% CI for the Youden index and its optimal cut-point value to provide highest sensitivity and specificity in the prediction of MACE for each strain parameter. Survival at 1 year after AMI was determined by Kaplan–Meier analysis for each 3D-STE parameter using each cut-off value. Considering the number of subjects in our pilot study (94 patients, 25 MACE), a maximum of 2 variables were included in the multivariate analysis. Hazard ratios (HR) and confidence intervals (CI) of parameters related to remodelling were estimated by Cox regression analysis. A post hoc power analysis was conducted based on the AUC for each cut-off value obtained for the 3D-STE parameters. Power analysis for survival rates (log rank test) between formed groups according to the cut-off values for each strain parameter was also performed based on the survival rate in each group, using an alpha of 0.05 and power of 80%.

Statistical analysis was performed using MedCalc Statistical Software 19.6.1 (MedCalc Software Ltd., Ostend, Belgium; http://www.medcalc.org; 2020 (accessed on 12 March 2021)). A *p* value of <0.05 was considered significant.

## 3. Results

### 3.1. Baseline Characteristics

Of the 110 patients, 7 were excluded due to improper quality of the images (suboptimal echocardiographic windows, inability to follow breath holding instructions, or presence of stitch artefacts), 3 patients were lost on follow-up, and 6 others were excluded due to death by non-cardiac cause.

**Figure 2:**
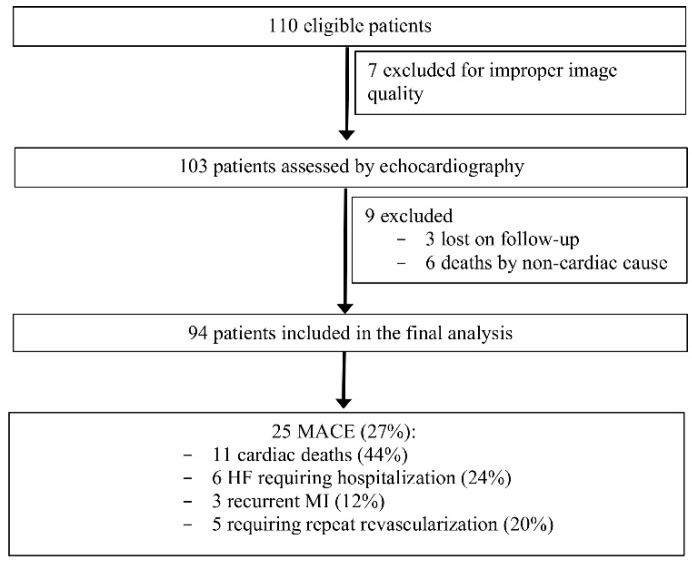
Study flowchart.

Therefore, 94 patients were included in the final analysis of this pilot study (Figure 2).

At the 1-year follow-up there were 25 MACE reported: 11 cases of cardiac death, 6 cases of HF requiring hospitalization, 3 cases with recurrent MI, and 5 cases requiring repeated revascularization. Baseline characteristics of patients with MACE (referred to as MACE+, *n =* 25, 27%) and without MACE (referred to as MACE−, *n* = 69, 73%) are summarized in Table 1. Patients with MACE presented more frequently with STEMI (*p* = 0.009) and multi-vessel CAD (*p* = 0.017) at admission. However, there was no difference regarding the infarct-related vessel between patients with or without MACE. Age and the presence of different cardiovascular risk factors were comparable between the two groups.

### 3.2. Standard and 3D Speckle-Tracking Echocardiography Parameters

Considering echocardiographic parameters, patients in MACE+ group showed significantly increased LV volumes and reduced LVEF (*p* = 0.007), but there was no difference considering diastolic dysfunction parameters and LV mass among groups. All 3D strain parameters (GLS-3D, GCS-3D, GAS, and GRS-3D) were significantly more impaired in MACE+ group, with the highest statistic significance for GCS-3D (*p* = 0.009) and GAS (*p* = 0.006), as shown in Table 1.

### 3.3. Impact of 3D Strain on the Occurrence of MACE at 1 Year after Acute Myocardial Infarction

**Figure 3:**
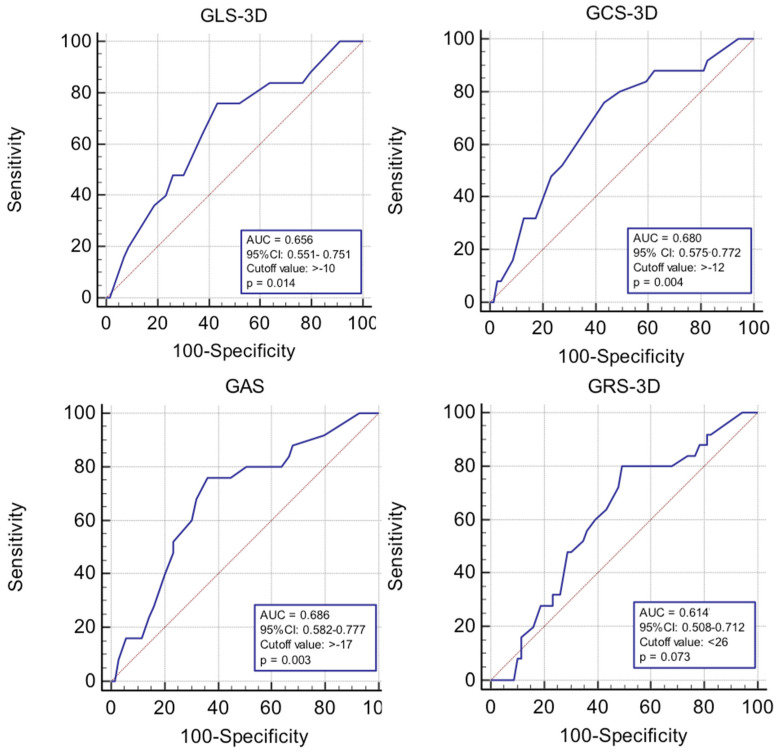
ROC curves used in the prediction of 1-year MACE after acute myocardial infarction; 3D, three-dimensional; AUC, area under the curve; CI, confidence interval; GAS, global area strain; GCS, global circumferential strain; GLS, global longitudinal strain; GRS, global radial strain; MACE, major adverse cardiovascular events; and ROC, Receiver Operating Characteristic.

We aimed to evaluate the impact of 3D strain parameters on the occurrence of MACE. Youden index of each of the four parameters was therefore determined by receiver operating characteristic curve analysis and the cut-off value obtained for each strain parameter was used to determine survival at 1 year (Figure 3).

**Figure 4:**
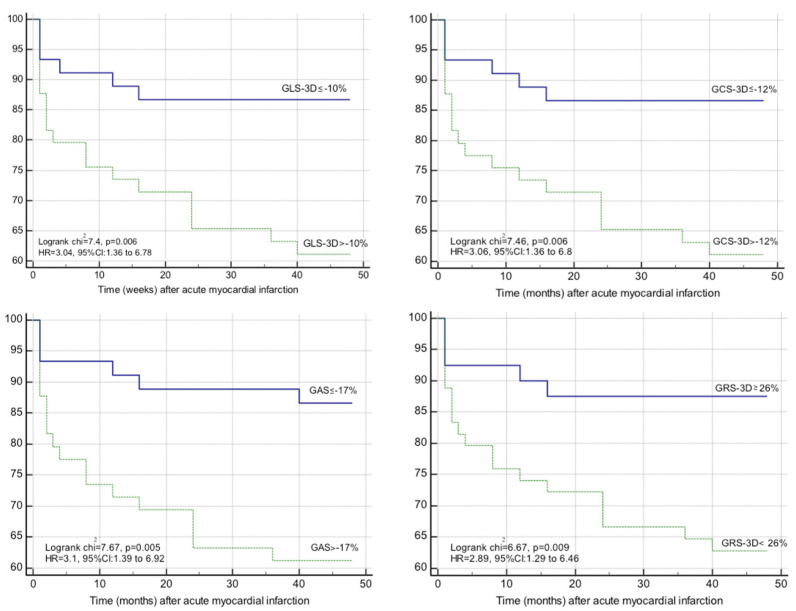
Kaplan–Meier survival analysis for 1-year MACE. Patients were stratified by best cut-off value derived from the Youden index; 3D, three-dimensional; GAS, global area strain; GCS, global circumferential strain; GLS, global longitudinal strain; GRS, global radial strain; and MACE, major adverse cardiovascular events.

Kaplan–Meier curves for event-free survival showed a significantly higher rate of MACE in patients with GLS-3D > −10% (logrank *p* = 0.006, HR = 3.04, 95% CI: 1.36–6.78), GCS-3D > −12% (logrank *p* = 0.006, HR = 3.06, 95% CI: 1.36–6.8), GAS > −17% (logrank *p* = 0.005, HR = 3.1, 95% CI: 1.39–6.92), and GRS-3D ≤ 25% (logrank *p* = 0.009, HR = 2.89, 95% CI: 1.29–6.46), as shown in Figure 4.

However, the multivariable models adjusted for each strain parameter and the number of affected vessels showed that only GAS (HR = 1.1, 95% CI: 1.03–1.16), GLS-3D (HR = 1.13, 95% CI: 1.03–1.26), and GCS-3D (HR = 1.13, 95% CI: 1.03–1.23) remained independent predictors of MACE, as seen in Table 2.

The post hoc power analysis for ROC curve analysis demonstrated that a total sample of 84 patients for GAS, 90 for GCS-3D, 120 for GLS-3D, and 228 for GRS-3D was required to achieve a power of 80%, showing an enough powered sample size only for GAS and GCS-3D. In the case of survival analysis, a total sample of 59 patients for GAS, 75 for GCS-3D, 112 for GLS-3D, and 75 for GRS-3D was required, showing insufficiently powered sample size for GLS-3D.

### 3.4. Interobserver Variability

Interobserver correlation coefficients for GLS-3D, GCS-3D, GRS-3D, and GAS were of 0.89, 0.92, 0.87, and 0.91, respectively. The observed variability was low, showing a good agreement between measurements in all 3D-STE parameters.

## 4. Discussion

Early estimation of the prognosis after AMI is important in daily clinical scenarios. As shown in other studies, although the entire spectrum of acute coronary syndromes (ACS) is a characteristic of the elderly population, over the past years there has been an increase in their incidence in young people [15]. LV systolic function remains the cornerstone in AMI patients’ evaluation and follow-up and LVEF is the most widely used parameter. However, LVEF has its limitations, caused mainly by load dependency and by the complex geometry of the LV. Studies conducted in the last decade demonstrated the necessity of intensive use of deformation parameters, which might detect more subtle changes in the LV function [4]. In contrast to 2D-STE, 3D-STE can simultaneously assess all the components of strain with a single post-processing algorithm [9] and might therefore provide a faster and more complete analysis of LV performance. However, 3D strain disadvantages are of being dependent of image quality and temporal and spatial resolution [9].

While several studies demonstrated the utility of 3D strain in various clinical scenarios [16,17,18], there are limited results reported regarding the clinical prognostic value of 3D-STE in patients after AMI. However, some recent studies showed that 3D strain might be useful in the prediction of LV remodelling and stratification of the risk in these patients [11,12,13,14,19].

We emphasize the need for assessment of multiple strain components in order to determine the impact of 3D-STE on MACE in patients with AMI, where geometric assumptions are required, and ischemia might involve multiple myocardial layers. The goal of our study was to determine the prognostic value of 3D-STE parameters in the prediction of clinical outcomes of patients with AMI undergoing successful PCI and we demonstrated that GLS-3D, GCS-3D, GAS, and GRS-3D were significantly associated with a 3-fold increase in the risk of MACE. However, among all 3D strain parameters, GAS, GLS-3D, and GCS-3D were the only independently associated with increased risk of MACE. GAS reflects the shortening of both longitudinal and circumferential layers from end-diastole at end-systole [20]. Therefore, it increases the magnitude of the change in deformation, and it is thought to better determine subtle LV function impairment.

As concerning the accuracy of 3-STE parameters, different studies reveal variability of the obtained values and their accuracy [19,20,21,22,23,24]. As for example, in the case of GLS-3D, Ivahashi et al. [13] found higher specificity (Sp of 84% and Se of 83%), while Sugano et al. [21] found lower sensitivity (Se of 47% and Sp of 83%) comparing with our study. As related to GAS, similar with our study, Ali et al. [12] also found a high sensitivity (87.5%) for values > −17%, while Sugano et al. [21] found both high sensitivity and specificity, but the values were significantly less impaired. However, that study evaluated the patients at only 6 days after PCI.

Although previous research showed that all 3D-STE parameters are predictors of MACE [12,14], GAS seems to be the most sensitive parameter in detecting early LV dysfunction in patients with AMI [11,12,14]. We found in our pilot study that the cut-off value of GAS showing highest sensitivity and specificity in the prediction of MACE was of −17%, which was associated with a 3.1-fold increase in MACE. A similar cut-off value was found to be predictive of clinical outcomes in patients with AMI in the research of Ali et al. [12]. Yet, Cai et al. found a less impaired value for GAS to predict MACE (of −21.5%), but this study evaluated the patients at one week after PCI and had a longer follow-up period (up to 3 years) [14].

Other studies proposed GLS as a predictor of LV remodelling and long-term MACE [5,22,23,24,25,26]. In our study, GLS-3D was also significantly higher in patients presenting MACE at 1 year. However, GLS-3D was also not as good as GCS-3D and GAS for the prediction of microvascular obstruction in two other studies [19,27] and similar results were obtained by Cai et al., who found that GRS-3D, GCS-3D, and GAS-3D were all significantly lower in patients with MACE after AMI, but only borderline significance was found for GLS-3D [14]. In contrast, Ivahashi et al. also found that GLS-3D was a predictor of remodelling and MACE at 1 year, but that study did not evaluate other 3D-STE parameters [13].

Even though the cut-off values for strain parameters were relatively low, there is a variability in the STE values reported in different studies. However, with the exception of Ivahashi et al. [13], which, similar to our case, also evaluated the patients at 48 h after PCI, the timing of echocardiographic measurements of the other studies [5,14,21] were different (Cai et al. at 1 week, Cimino et al. at 6 ± 2 days, and Sugamo et al. 6 days), and it is well-known that LV deformation parameters might vary in the following days after PCI. We believe that these differences among studies are due to the timing of evaluation and due to the patients’ backgrounds.

As related to other echocardiographic parameters, we found no difference in diastolic dysfunction parameters in patients with MACE, and these results are similar to those of Ivahashi et al., who also found an association between MACE and 3D-STE, but not with E/e’ [13]. This might be explained by the change of tissue Doppler indices values in the acute setting and the need of assessing them only in the stable phase of AMI.

Regarding clinical factors, although multivariate analysis did not demonstrate an independent association with MACE, we identified that the number of affected vessels and eGFR were the only clinical variables to be associated with MACE on the univariate regression. This associations have been documented by a previous study revealing that triple vessel disease is associated with a 4-fold increase in MACE, and eGFR is also a predictor of events [28]. However, the same research found that arterial hypertension was also an independent predictor of MACE after AMI. In our case, the lack of association between arterial hypertension and MACE might be explained by the presence of this cardiovascular risk factor in the majority of the patients with AMI. Another study found that, among all clinical variables, the only one associated with MACE was the anterior location of AMI. The same study demonstrated that a model including both infarct location and GAS showed the highest discriminative capacity in the prediction of MACE [14]. However, two other studies also demonstrated that cardiovascular risk factors and serum biomarkers are not correlated to the occurrence of MACE. Moreover, the same two studies did not find the location of AMI to be a predictor of MACE either [12,29]. We also found an increased rate of MACE events in women. These results are similar to those of other recent studies, which show higher incidence of MACE in women than in men, mainly due to poorer cardiovascular risk factor profile and older age [30], but also due to increased bleeding risk [31]. Overall, mortality rates were higher in our study compared to those reported in Western Europe. A recent report aiming to assess mortality rates after acute myocardial infarction in Romania between 1994–2017 showed that, although there was a decrease in the mortality rate after AMI, mortality remains increased as compared to Western Europe. The explanation was the more difficult access to PCI centres and the high prevalence of cardiovascular risk factors in Romanian population (sedentary lifestyle, cigarette smoking arterial hypertension, obesity, metabolic syndrome, and diabetes) [32].

The feasibility of 3D-STE is influenced by several factors. One of the major concerns of 3D-STE is the balance between temporal and spatial resolution, as higher frame rates may lead to some compromise in spatial resolution. Therefore, the accuracy of 3D-STE depends on optimal image quality, limiting the number of patients in whom 3D-STE is feasible in clinical practice. A stable image is important in order to obtain high temporal and spatial resolution and to avoid stitch artefacts. Thus, the acquisition is vulnerable to motion artefacts if the patient is unable to follow breath hold instructions. These limitations might occur in the setting of AMI, where temporal resolution and breath holding are more challenging [9,33]. Reproducibility of 3D-STE has been reported as acceptable to excellent in several studies [34,35]. In our study, variability between measurements was also low. The highest variability was reported for GRS-3D, while GAS showed the best reproducibility among all 3D-STE parameters. Variability for 3D-STE might be caused by factors influencing temporal resolution, such as the acquisition, post-processing, and haemodynamic status of the patient. However, 3D-STE remains a time saving echocardiography technique, as it allows the calculation of all strain parameters from a single data set and also avoids errors caused by irregular heart rhythms.

### Limitations

Our study has several limitations. It was a single-centre study and included a small population. While the post hoc power analysis indicated adequate sample size for the determination of the cut-off value to predict MACE in the case of GAS and GCS-3D and also for the survival analysis in the case of GRS-3D, more patients would be needed for a sufficiently powered sample size in the case of GLS-3D and GRS-3D. Follow-up duration was relatively short and all measurements were performed in the acute setting, with no follow-up data of echocardiographic measurements. At the same time, as it is well-known that the short- and long-term survival rates are different in STEMI and NSTEMI, the impact of 3D-STE on MACE according to the type of AMI might be the subject of a future, larger study. In addition, compared to 3D strain, the prognostic value of 2D-STE was already demonstrated by multiple studies, raising the question whether 3D-STE might bring additional benefits to 2D deformation parameters. Therefore, further studies should use longer follow-up periods with larger number of patients and should compare the accuracy of 3D-STE and 2D-STE in the prediction of MACE.

## 5. Conclusions

Our study demonstrated that 3D strain parameters, particularly GAS and GCS-3D, are associated to an increased risk of 1-year MACE after AMI managed by primary PCI. Thus, with the aid of baseline echocardiographic examinations, patients might benefit from early risk stratification.

## Figures and Tables

**Table 1:** Baseline characteristics of patients with and without MACE.

	All Patients (*n* = 94)	MACE− (*n* = 69)	MACE+ (*n* = 25)	*p*
Clinical characteristics				
Age, mean (SD), years	66 (13)	66 (13)	69 (13.5)	NS
Male gender, n (%)	53 (56)	42 (60)	11 (44)	<0.001
BMI, kg/m^2^	29 (5)	29 (4.8)	29 (5.8)	NS
Current smoking, n (%)	38 (40)	30 (43)	8 (32)	NS
Diabetes mellitus, n (%)	33 (35)	24 (33)	7 (28)	NS
Hypertension, n (%)	73 (77)	53 (77)	20 (80)	NS
Dyslipidaemia, n (%)	26 (28)	17 (25)	9 (35)	NS
Heart rate, mean (SD), bpm	79 (18)	78 (17)	80 (21)	NS
Systolic blood pressure, mean (SD), mmHg	132 (26)	135 (24)	124 (29)	NS
Diastolic blood pressure, mean (SD), mmHg	76 (15)	77 (14)	72 (16)	NS
STEMI/NSTEMI, n (%)	65 (69)/29 (31)	46 (66)/23 (44)	19 (76)/6 (24)	0.009
Biomarker levels				
Troponin T, median (IQR), ng/mL	0.29 (0.11–1.02)	0.30 (0.09–0.93)	0.28 (0.11–1.66)	NS
eGFR, mean (SD), mL/min/1.73 m^2^	91 (40)	97 (35)	75 (48)	0.02
Glucose, mean (SD), mg/dL	136 (49)	133 (44)	145 (60)	NS
Total cholesterol, mean (SD), mg/dL	184 (52)	187 (53)	178 (49)	NS
High-density lipoprotein, mean (SD), mg/dL	43 (12)	43 (11)	43 (13)	NS
Low-density lipoprotein, mean (SD), mg/dL	111 (46)	111 (47)	108 (46)	NS
Triglycerides, mean (SD), mg/dL	155 (81)	157 (78)	150 (92)	NS
Coronary artery characteristics				
Infarct related artery, n (%)				
LAD	49 (52)	34 (49)	15 (60)	NS
CX	14 (15)	13 (17)	1 (4)	
RCA	31 (33)	22 (32)	9 (36)	
Coronary artery disease, n (%)				
1-vessel	43 (46)	38 (55)	5 (20)	0.017
2-vessel	27 (29)	15 (22)	12 (48)	
3-vessel	24 (25)	16 (23)	8 (32)	
Echocardiography				
LV EDV, mean (SD), mL	117 (38)	110 (32)	134 (47)	0.006
LV ESV, mean (SD), mL	67 (13.3)	60 (29)	84 (43)	0.002
LV mass, mean (SD), g	104 (13.5)	103 (13.6)	108 (13)	NS
LVEF, mean (SD), %	45 (12)	45 (11)	38 (10)	0.007
E/e’, mean (SD)	11 (6)	10.4 (5)	11.6(8)	NS
LAVi, mean(SD), mL/m^2^	29.4 (7)	30 (7)	30 (7)	NS
vmaxTR, mean (SD), m/s	2.4 (0.9)	2.4 (0.9)	2.5 (0.8)	NS
GLS-3D, mean (SD), %	−9.5 (4.4)	−10.1 (4.4)	−7.8 (4)	0.02
GCS-3D, mean (SD), %	−11.7 (5.1)	−12.5 (5.2)	−9.5 (4.5)	0.009
GAS, mean (SD), %	−17.7 (7.4)	−19 (7.4)	−14.2 (6.4)	0.006
GRS-3D, mean (SD), %	26.8 (16)	28.6 (17)	21 (12.4)	0.04

3D, three-dimensional; BMI, body mass index; CX, circumflex; EDV, end-diastolic volume; ESV, end-systolic volume; GAS, global area strain; GCS, global circumferential strain; GLS, global longitudinal strain; GRS, global radial strain; LAD, left anterior descending; LVEF, left ventricular ejection fraction; LAVi, left atrial volume index; MACE, major adverse cardiovascular events; NSTEMI, non-ST-elevation myocardial infarction; RCA, right coronary artery; SBP; systolic blood pressure; STEMI, ST-elevation myocardial infarction; and TR, tricuspid regurgitation.

**Table 2:** Cox regression analysis between studied parameters and MACE.

Parameter	Univariate Analysis		Multivariate Analysis	
	Unadjusted HR (95% CI)	*p*	Adjusted HR (95% CI)	*p*
GLS-3D, %	1.11 (1.01–1.23)	0.02	1.13 (1.03–1.26)	0.02
GCS-3D, %	1.11 (1.02–1.21)	0.01	1.13 (1.03–1.23)	0.01
GAS, %	1.08 (1.02–1.14)	0.007	1.1 (1.03–1.16)	0.005
GRS-3D, %	0.97 (0.94–1)	0.09		
Age, years	1.01 (0.99–1.04)	0.27		
Male gender	0.56 (0.25–1.24)	0.17		
BMI, kg/m^2^	1 (0.93–1.09)	0.9		
SBP, mmHg	1 (0.98–1)	0.07		
eGFR, mL/min/1.73 m^2^	0.98 (0.97–1)	0.01		
STEMI/NSTEMI	0.66 (0.26–1.65)	0.37		
Number of vessels	1.68 (1.07–2.6)	0.02		
LV mass, g	1.02 (0.99–1.05)	0.14		
LVEF, %	0.95 (0.96–0.99)	0.006		
E/e’,	1.03 (0.97–1.1)	0.32		
LAVi, mL/m^2^	1.03 (0.97–1.1)	0.32		
Vmax TR, m/s	1.12 (0.78–1.62)	0.54		

Regression Analysis—all cases (*n* = 94, 25 MACE): adjusting for each strain parameter + number of diseased vessels; 3D, three-dimensional; BMI, body mass index; GAS, global area strain; GCS, global circumferential strain; GLS, global longitudinal strain; GRS, global radial strain; LVEF, left ventricular ejection fraction; LAVi, left atrial volume index; MACE, major adverse cardiovascular events; NSTEMI, non-ST-elevation acute myocardial infarction; SBP, systolic blood pressure; STEMI, ST-elevation acute myocardial infarction; and TR, tricuspid regurgitation.

## Data Availability

The data supporting the findings of this study are available upon request from the corresponding author.
